# Dendritic Cells from Aged Subjects Display Enhanced Inflammatory Responses to *Chlamydophila pneumoniae*

**DOI:** 10.1155/2014/436438

**Published:** 2014-09-01

**Authors:** Sangeetha Prakash, Sudhanshu Agrawal, Dandan Ma, Sudhir Gupta, Ellena M. Peterson, Anshu Agrawal

**Affiliations:** ^1^Division of Basic and Clinical Immunology, Department of Medicine, University of California, Irvine, CA 92697, USA; ^2^Department of Pathology and Laboratory Medicine, University of California, Irvine, CA 92697, USA

## Abstract

*Chlamydophila pneumoniae* (CPn) is a common respiratory pathogen that causes a chronic and persistent airway infection. The elderly display an increased susceptibility and severity to this infection. However, the underlying mechanisms are not well understood. Dendritic cells (DCs) are the initiators and regulators of immune responses. Therefore, we investigated the role of DCs in the age-associated increased CPn infection *in vitro* in humans. Though the expression of activation markers was comparable between the two age groups, DCs from aged subjects secreted enhanced levels of proinflammatory mediators such as TNF-*α* and CXCL-10 in response to CPn. In contrast, the secretion of IL-10 and innate interferons, IFN-*α* and IFN-*λ*, was severely impaired in DCs from aged donors. The increased activation of DCs from aged subjects to CPn also resulted in enhanced proliferation of CD4 and CD8 T cells in a DC-T coculture. Furthermore, T cells primed with CPn-stimulated DCs from aged subjects secreted increased levels of IFN-*γ* and reduced levels of IL-10 compared to DCs obtained from young subjects. In summary, DCs from the elderly displayed enhanced inflammatory response to CPn which may result in airway remodeling and increase the susceptibility of the elderly to respiratory diseases such as asthma.

## 1. Introduction

Elderly individuals are at high risk to develop chronic airway inflammatory illnesses such as asthma and chronic obstructive pulmonary disease (COPD) [1–3]. Among all airway illnesses, asthma is emerging as one of the most important and prevalent diseases [3]. A recent survey estimates that asthma affects 4–8% of the American population over 65 years of age and numbers are expected to increase in the upcoming years [3]. Most deaths caused by asthma also occur in this age group [2, 3]. Acute respiratory viral infections such as influenza are often the trigger for asthma attacks and therefore considered the primary culprits in the pathogenesis of asthma in the elderly [4, 5].

There is a scarcity of information about the influence of chronic or latent respiratory infections, which could induce prolonged airway inflammation.* Chlamydophila pneumoniae* (CPn) is an obligate intracellular bacterial pathogen that causes chronic, persistent, and often asymptomatic infections [6]. It is also reported to be implicated in exacerbation of asthma, pharyngitis, and bronchitis [7]. Epidemiological data suggest that most people are infected and reinfected throughout life [8]. Community-acquired pneumonia (CAP) remains a significant cause of morbidity and death worldwide and a significant threat to the elderly despite the availability of effective antibiotics. CAP due to atypical pathogens, CPn, and* Mycoplasma pneumoniae*, as single or copathogens, can cause severe diseases in older patients, resulting in hospitalization. Studies from the literature suggest that* Mycoplasma* and* Chlamydophila* are responsible for 9–12% and 6–13% of CAP, respectively [9]. Reports also indicate that the prevalence of antibodies specific to CPn increases significantly in the elderly [10]. Furthermore, aged mice infected with CPn displayed an increased severity of infection compared to young animals [11]. The age-associate alterations in the function of immune system that may play a role in the increased incidence and severity of CPn infections in the elderly are not well understood.

Dendritic cells (DCs) are present below the epithelial cells lining the airway and are amongst the primary responders to airway infections [12]. DCs present at the airways sense and capture pathogens through a variety of pathogen recognition receptors (PRRs). Subsequently DCs become activated by upregulating the expression of costimulatory and antigen-presenting molecules as well as secreting proinflammatory cytokines. During activation, DCs migrate to the draining lymph nodes to prime and activate naïve T cells. The activation molecules and cytokines secreted by DCs have a major influence on T cell responses [13, 14]. We and others have reported that advancing age significantly impacts DC functions and alters their response to infections [15]. DCs from aged donors exhibit enhanced inflammatory responses at the basal level and display increased reactivity to self-antigens [16, 17]. In contrast to self-antigens, the response of DCs from elderly to pathogens is often compromised. For example, DCs from aged subjects are severely impaired in their capacity to produce interferon-*α* (IFN-*α*) and interferon-*λ* (IFN-*λ*) after activation with influenza virus [18, 19]. Recent* in vivo* studies in mice have demonstrated that CPn can infect DCs and alter their function to induce a proinflammatory state. Furthermore, CPn may also utilize DCs for dissemination outside the lung [20, 21]. Age-associated alterations in the response of DCs to CPn may therefore play a major role in the increase in CPn infections in the elderly.

The objective of the present study was to compare the response to CPn of DCs obtained from both young and elderly subjects.

## 2. Material and Methods

### 2.1. Blood Donors

Blood samples, donated by healthy elderly and young volunteers, were collected. The age of young donors was between 20 and 35 years and that of elderly was between 65 and 90 years. Elderly subjects were considered middle class in terms of socioeconomic status and living independently. The description of the cohort is provided in Table 1. This study was approved by the Institutional Review Board of the University of California (Irvine, CA, USA).

### 2.2. *In Vitro* Derivation and Culture of Human Monocyte-Derived DCs

DCs were prepared as described before [22]. Peripheral blood mononuclear cells (PBMCs) were separated by Ficoll-Hypaque density gradient centrifugation. Monocytes were purified from the PBMCs by positive selection with anti-CD14 microbeads (StemCell Sep, Vancouver, BC, Canada). The purity of the isolated CD14^+^ monocytes was >90%, as determined by flow cytometry. For the induction of DC differentiation, purified CD14^+^ monocytes were cultured in a humidified atmosphere of 5% CO_2_ at 37°C in RPMI 1640 supplemented with 10% FBS, 1 mM glutamine, 100 U/mL penicillin, 100 *μ*g/mL streptomycin, 50 ng/mL human rGM-CSF (PeproTech, Rocky Hill, NJ, USA), and 10 ng/mL human rIL-4 (PeproTech). Half of the medium was replaced every 2 days and DCs (CD14^−^HLA-DR^+^CD11c^+^ cells) were collected after 6 days. The purity of the DCs was >95% as determined by the expression of CD14, CD11c, and HLA-DR.

### 2.3. Stimuli

*C. pneumoniae* CM-1 (ATCC, Manassas, VA, USA) was propagated in Hep-2 cells and stored in 2-sucrose phosphate glutamate buffer at −80°C. Immature DCs collected on day 6 were stimulated with UV inactivated* CPn* with an MOI equivalent to 0.01 CPn : 1 DC or 0.1 CPn : 1 DC or 1CPn : 1 DC.

### 2.4. DC Phenotype

Control and CPn-stimulated DCs from both age groups of subjects were stained for the surface expression of CD80, CD86, CD83, and HLA-DR using directly conjugated Abs (BD Pharmingen, San Diego, CA, USA). A total of 10,000 CD14^−^CD11c^+^ cells per condition were acquired using a FACSCalibur (BD Pharmingen). Data analysis was performed using a FlowJo (Tree Star Inc., Ashland, OR, USA).

### 2.5. Cytokine and Chemokine Production by DC

DCs were stimulated with CPn for 24 h as described above. Supernatants were collected and stored at −70°C until being analyzed. Cytokines, IFN-*α*, IL-6, TNF-*α*, IL-10, IL-1*β*, CXCL-8, CXCL-10, and IL-12p40 in the supernatants were measured by Flow Cytomix (BD Pharmingen) as per the manufacturer's protocol. IFN-*λ* was measured using an ELISA (PBL Biomedicals, Piscataway, NJ, USA).

### 2.6. Allogeneic DC T Cell Cocultures

Immature DCs were stimulated with CPn as described above. After 24 h of incubation, cells were collected and washed. Subsequently, 1 × 10^4^ DCs were cultured with 1 × 10^5^ magnetic bead purified (StemCell, Vancouver, BC, Canada), naïve allogeneic T cells from young individuals. For naïve T cell isolation, first the total T cells were isolated by negative selection; subsequently, CD45RO beads were used to remove the memory cells. The remaining cells were >95% CD45RA^+^ as determined by flow cytometry. DCs from aged and young subjects for each experiment were cultured with allogeneic T cells from one young subject for comparison. The T cells were from a different young subject for each experiment. After 7 days of incubation, the supernatant was collected and the secretion of IFN-*γ*, IL-10, IL-21 (eBiosciences, San Diego, CA, USA), IFN-*γ* (BD Pharmingen), and IL-17 (eBiosciences) was assessed using ELISA.

### 2.7. CD4 and CD8 T Cell Proliferation

Stimulated DCs were cultured with purified, CFSE-labeled allogenic T cells from young donors at a ratio of 1 : 10 in 96-well plates. The purity of T cells recovered from DC T cell cultured after 7 days was 85–91%, as determined by flow cytometry. Cells were then collected and stained for CD4 and CD8 markers. By measuring the dilution of CFSE, we determined the proliferative capacity of gated CD4 T and CD8 T cells. Proliferation index (total number of divisions divided by the number of cells that went into division) was calculated using the proliferation platform of FlowJo.

### 2.8. Isolation and Culture of Human Conventional Myeloid DCs (cDCs) from Blood

cDCs were enriched from the PBMCs of aged and young subjects by negative selection using magnetic beads based kit from StemCell Separation. Purity was >90% as determined by flow cytometry using CD11c and HLADR. Approximately, 1–3 × 10^5^ cDCs were recovered from 40 mL of blood. cDCs (5 × 10^5^/mL) were stimulated with UV inactivated CPn with a MOI equivalent to 1 DC : 1 CPn for 24 h. Supernatants collected were assayed for various cytokines as described above for DCs.

### 2.9. Statistical Analysis

Data were analyzed and figures were generated using GraphPad Prism 5.00 software (GraphPad Software, San Diego, USA). Significant differences between groups were determined by Mann-Whitney test at 90% confidence interval (*P* value < 0.05 was considered significant).

## 3. Results

### 3.1. Comparable Expression of Activation Markers in DCs from Aged and Young Subjects in Response to CPn

Advancing age may alter the response of DCs to CPn [11]. Therefore, we investigated whether the upregulation of DC activation markers in response to CPn was altered in DCs from aged subjects as compared to DCs from young subjects.

Subjects are described in Table 1. The control population included 24 individuals in the age range of 20–32 with an average age of 26. The geriatric population consisted of 24 individuals in the age range of 65–93 with an average age of 78. The younger individuals were healthy and not on medications.

**Figure 1:**
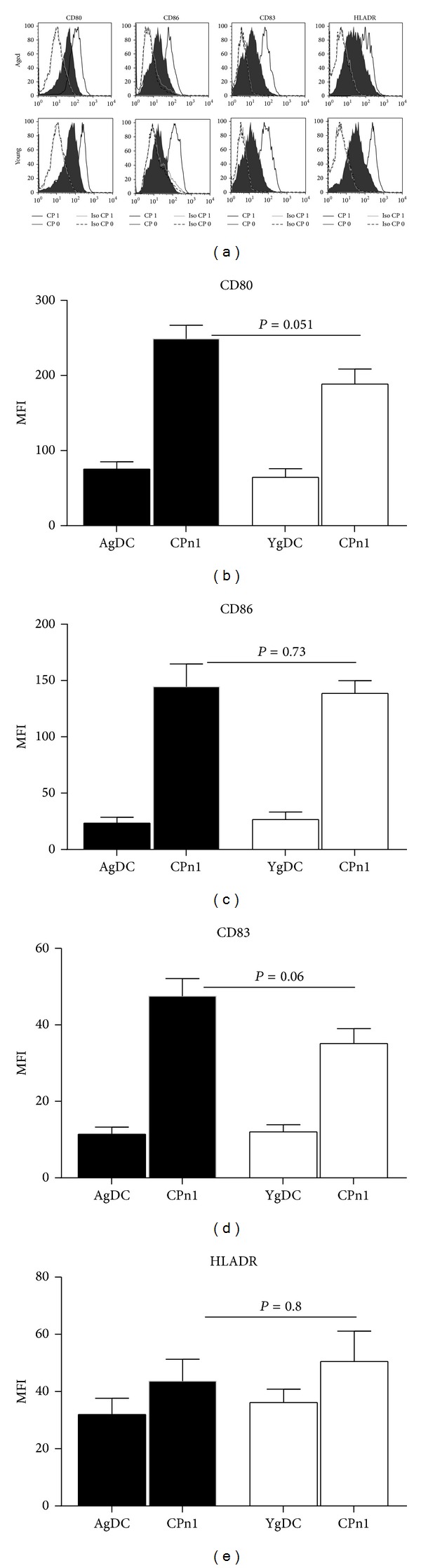
Comparable expression of activation markers in DCs from aged and young subjects in response to CPn. (a) Histograms depict the expression of activation markers on DCs from aged and young subjects after on stimulation with CPn. Bar graphs depict the MFI of the expression of activation molecules on CPn stimulated DCs from aged and young subjects. (b) CD80; (c) CD86; (d) CD83; (e) HLADR. Data is mean +/− S.E of 12 aged and 12 young subjects. *P* value depicted is comparison of CPn-stimulated DCs from aged subjects with their young counterparts.

DCs from aged and young individuals were stimulated with UV inactivated CPn at a ratio of 1 : 1 for 24 h. This concentration of CPn was found to be optimal (Supplementary Figure  1 in Supplementary Material available online at http://dx.doi.org/10.1155/2014/436438). Stimulation with the bacteria resulted in substantial activation of DCs from both aged and young subjects (Figure 1). The expression of activation markers was comparable on DCs from aged and young subjects (Figure 1(a)). Average of mean fluorescent intensity (MFI) of all subjects suggested that DCs from aged subjects displayed enhanced expression of CD80 and CD83 but the difference was not significant (Figures 1(b) and 1(d)). The MFI of expression of CD86 and HLA-DR on the other hand was comparable in DCs obtained from both age groups (Figures 1(a), 1(b), and 1(e)). This data suggest that infection with CPn leads to slight though insignificant increase in activation and maturation of DCs from aged as compared to DCs from young subjects.

### 3.2. Altered Secretion of Cytokines and Chemokines by DCs from Aged Donors as Compared to DCs from Young Donors after Stimulation with CPn

**Figure 2:**
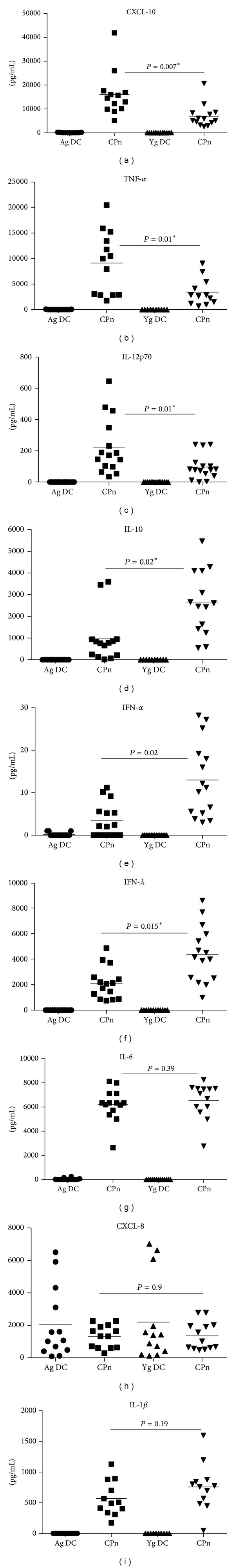
Altered secretion of cytokines and chemokines by DCs from aged subjects as compared to DCs from young subjects after stimulation with CPn. Graphs depict the levels of cytokine and chemokines secreted by CPn-stimulated DCs from aged and young subjects. (a) CXCL-10; (b) TNF-*α*; (c) IL-12; (d) IL-10; (e) IFN-*α*; (f) IFN-*λ*; (g) IL-6; (h) CXCL-8; (i) IL-1*β*. Each dot corresponds to a separate subject. Data is of 13 aged and 13 young subjects. *P* value depicted is comparison of CPn-stimulated DCs from aged subjects with their young counterparts.

Next, we investigated the cytokines secreted by these stimulated DCs. After stimulation with CPn for 24 h, supernatants were collected and assayed with Flow Cytomix and/or ELISA to quantify cytokine secretion. Stimulation of DCs with CPn induced the production of increased levels of proinflammatory cytokines (IL-6, TNF-*α*, IL-1*β*, IL-12, IFN-*α*, and IFN-*λ*), chemokines (CXCL-8, CXCL-10), and anti-inflammatory cytokine (IL-10) over unstimulated DCs in both age groups (Figure 2). However, the level of the secreted cytokines and chemokines was substantially different between the two populations. Stimulation with CPn resulted in significantly enhanced secretion of CXCL-10 (*P* = 0.007, Figure 2(a)) and TNF-*α* (*P* = 0.01, Figure 2(b)) in DCs from aged subjects as compared to DCs from young subjects. Similar to the Th1 favoring chemokine CXCL-10, DC from aged donors also secreted significantly higher levels of IL-12 relative to DCs from young donors (*P* = 0.01, Figure 2(c)). In contrast, secretion of anti-inflammatory cytokine, IL-10 was significantly reduced in CPn-stimulated DCs from aged subjects compared to DCs from young subjects (*P* = 0.02, Figure 2(d)). Interestingly this high secretion of IL-10 by DCs from aged subjects was also observed when DCs from aged and young subjects were incubated with a lower number of CPn, that is, 0.1 CPn and 1 CPn (data not shown), implying that exposure to CPn, even at lower concentrations, induces an immunosuppressive response rather than an inflammatory response in DCs from young subjects. However, in DCs derived from aged individuals, this response is reversed and is more of an inflammatory response.

Previous studies have reported a substantial decline in the production of innate interferons, IFN-*α* and IFN-*λ*, by DCs from aged donors in response to respiratory viral infections [18, 19, 23]. Therefore, we investigated whether this deficiency in IFN production extends to respiratory bacterial infections. Similar to our results with viral infections, incubation of DCs from aged subjects with CPn resulted in significantly reduced levels of IFN-*α* (*P* = 0.02, Figure 2(e)) and IFN-*λ* (*P* = 0.015, Figure 2(f)) relative to DCs from young subjects. The secretion of all other cytokines, IL-6, CXCL-8, and IL-1*β* (Figures 2(g)–2(i)), was comparable in CPn-stimulated DCs from both groups. In summary, these pieces of data demonstrate that advancing age enhances the capacity of CPn-stimulated DCs from aged to secrete proinflammatory and Th1 promoting cytokines and chemokines which is accompanied by reduced secretion of anti-inflammatory and protective cytokines.

In the aged subjects there were several subgroups based on comorbidities (Table 1). Subgroup analysis was performed for osteoarthritis, hypertension, and subjects taking vitamins and antioxidants as these subgroups had sufficient subject numbers. However, we did not observe any significant difference in any of the cytokine levels between the two groups (*P* > 0.5). Based on these subgroup analyses, we feel fairly confident that the comparisons between the young control and aged subject populations are yielding valid results across a general geriatric group.

### 3.3. Conventional Myeloid DCs (cDCs) from the Blood of Aged Subjects Also Display Enhanced Inflammatory Responses to CPn

**Figure 3:**
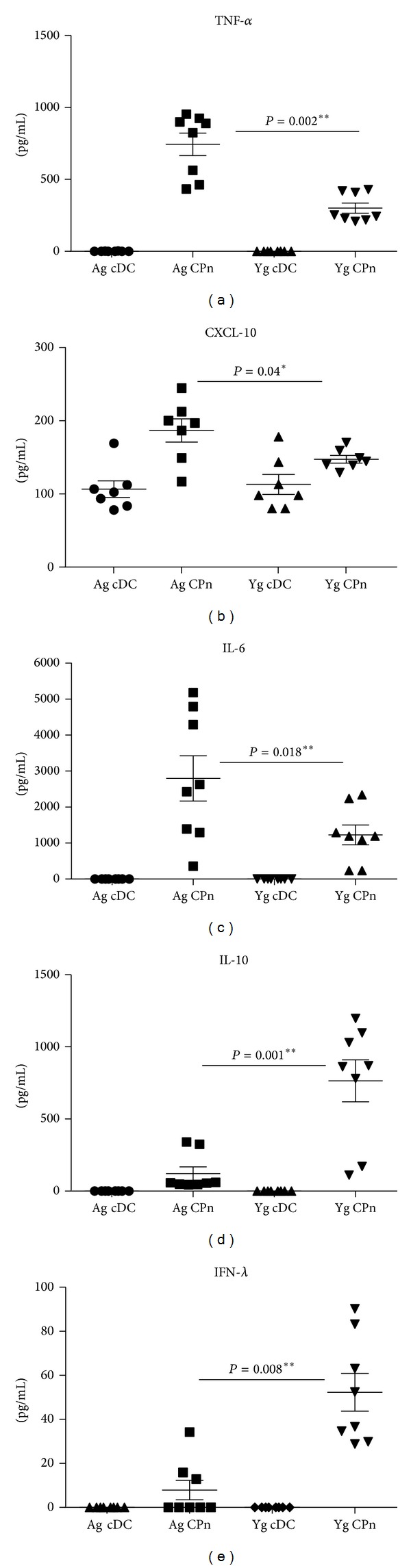
Conventional Myeloid DCs (cDCs) from the blood of aged subjects also display enhanced inflammatory responses to CPn. Graphs depict the levels of cytokine and chemokines secreted by CPn-stimulated cDCs purified directly from the blood of aged and young subjects. (a) TNF-*α*; (b) CXCL-10; (c) IL-6; (d) IL-10; (e) IFN-*λ*; Each dot corresponds to a separate subject. Data is of 8 aged and 8 young subjects. *P* value depicted is comparison of CPn-stimulated DCs from aged with their young counterparts.

Experiments described above were performed with DCs derived from monocytes* in vitro* in the presence of rGM-CSF and rIL-4. To rule out any possible artifacts in the response of DCs due to* in vitro* derivation, we investigated the response of directly purified cDCs from the blood of aged and young subjects to CPn. Similar to monocyte derived DCs, stimulation with CPn resulted in significantly enhanced secretion of TNF-*α* (*P* = 0.002, Figure 3(a)) and CXCL-10 (*P* = 0.04, Figure 3(b)) by cDCs from aged subjects relative to cDCs from young subjects. The secretion of IL-6 was also significantly higher (*P* = 0.018, Figure 3(c)) in cDCs from aged subjects compared to their young counterparts. In contrast, cDCs from aged donors displayed significant impairment in the production of IL-10 (*P* = 0.001, Figure 3(d)) and IFN-*λ* (*P* = 0.008, Figure 3(e)) relative to cDCs from young donors. The levels of IL-12, IL-1*β*, CXCL-8, and IFN-*α* were below the detection limits of our assay. These pieces of data suggest that both* in vitro* derived DCs and cDCs from the blood of aged subjects display similar responses to CPn stimulation. The rest of the experiments were therefore performed with* in vitro* derived DCs.

### 3.4. Enhanced CD4 and CD8 T Cell Proliferation by CPn-Stimulated DCs from Aged Donors

In order to determine whether the T cell priming capacities of CPn-stimulated DCs are also altered in the elderly, we investigated the capacity of DCs from aged and young subjects to induce the proliferation of CD4 T and CD8 T cells utilizing an allogeneic DC-T cell coculture system described previously [22]. CPn-activated and nonactivated DCs were cultured with naïve, purified, CFSE-labeled T cells from young, healthy individuals (to rule out T cell abnormalities) as the T cells from the elderly have been reported to have numerous defects [24, 25]. Six days later, T cells were assayed for proliferation by measuring the dilution of CFSE dye by flow cytometry.

**Figure 4:**
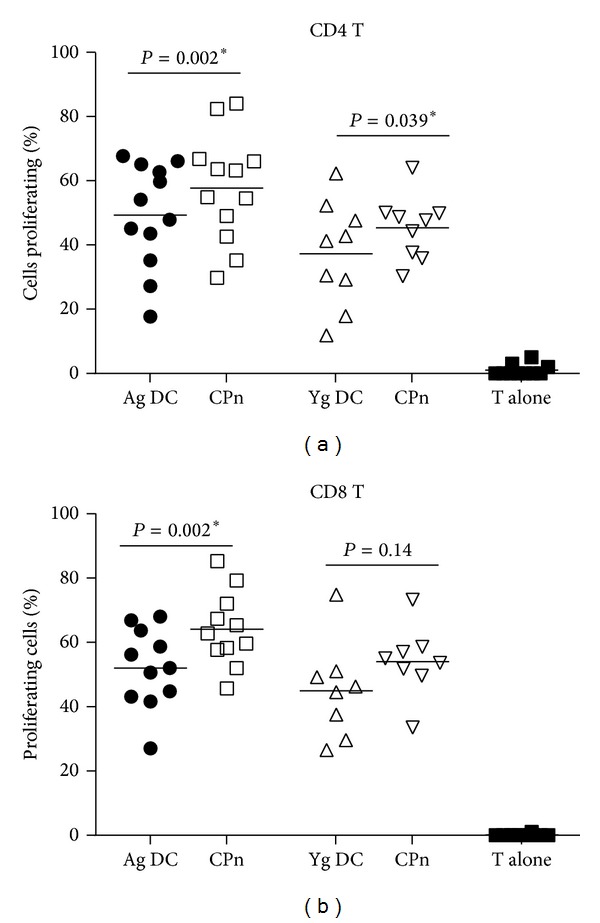
Enhanced proliferation of CD4 and CD8 T cells by CPn-stimulated DCs from aged subjects in an allogeneic DC-T cell coculture. CPn-stimulated and CPn-unstimulated DCs from aged and young subjects were cultured with allogeneic, CFSE labeled T cells from young subjects for 6 days. Graphs depict the proliferation of (a) CD4 T cells; (b) CD8 T cells as determined by flow cytometry. Each dot corresponds to a separate subject. Data is of 12 aged and 9 young subjects for (a) and 11 aged and 8 young for (b).  *P* value depicted is comparison of CPn-stimulated DCs with unstimulated DCs for both aged and young subjects.

Culture of CD4 T cells with CPn-stimulated DCs from aged subjects induced approximately 58% proliferation in CD4 T cells relative to unstimulated DCs which induced 47% proliferation (Figure 4(a), *P* = 0.002). CPn-stimulated DCs from young subjects also induced the significant level of proliferation of CD4 T cells (45%) over unstimulated DCs (38%) (Figure 4(a), *P* = 0.039). Therefore, the increase in CD4 T cell proliferation was 4% greater than that in coculture of DCs from aged and T cells (Figure 4(a)).

Proliferation of CD8 T cells followed a similar pattern with significantly increased CD8 T cell proliferation being observed after culture with CPn-stimulated DCs from aged individuals (65%) (Figure 4(b),  *P* = 0.002) relative to unstimulated DCs (52%). In contrast, CPn-stimulated DCs from young subjects failed to enhance significantly increased CD8 T cells proliferation (49%) relative to unstimulated DCs (54%) (Figure 4(b), *P* = 0.14). No proliferation was observed in both CD4 and CD8 T cells cultured alone without DCs. Altogether, these pieces of data suggest that DCs from aged subjects induce a more vigorous T cell response against CPn infection compared to DCs from young subjects.

### 3.5. CPn-Stimulated DCs from Aged Donors Induce Enhanced Levels of Proinflammatory Cytokines in T Cells

**Figure 5:**
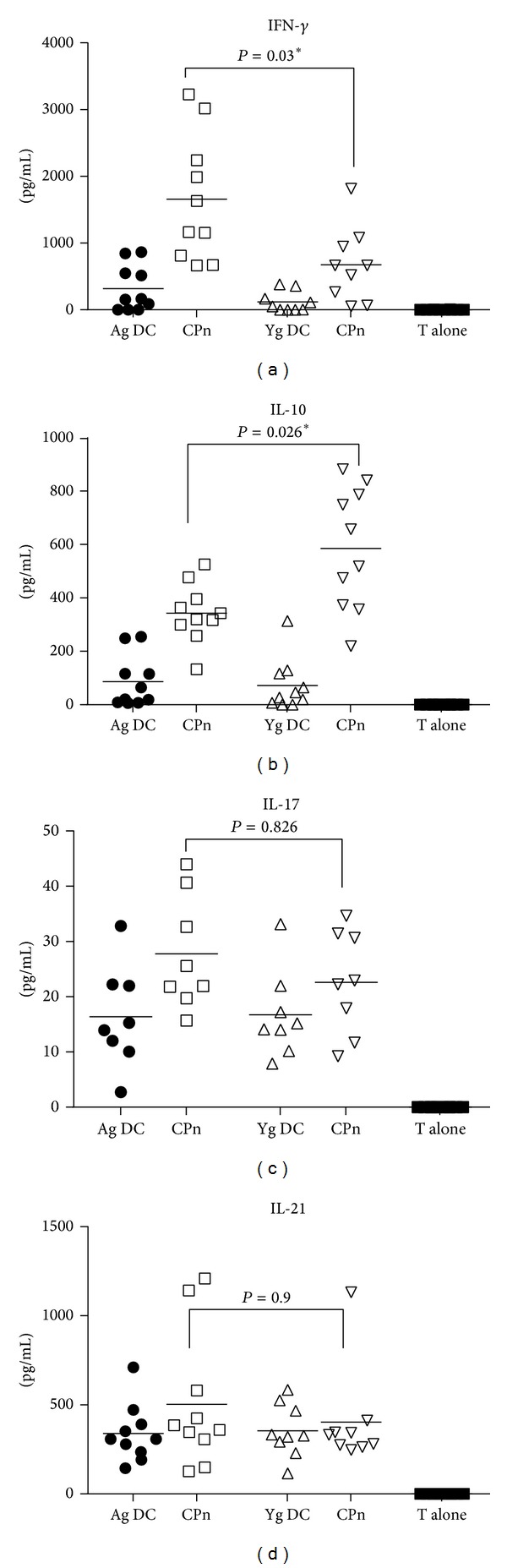
Effect of CPn-stimulated DCs from aged and young donors on T cell cytokine induction. Graphs depict the level of cytokines secreted by T cells after 6 days in an allogeneic coculture with CPn-stimulated DCs from aged and young subjects. (a) IFN-*γ*; (b) IL-10; (c) IL-17; (d) IL-21. Each dot corresponds to a separate subject. Data is of 10 aged and 10 young subjects. *P* value depicted is comparison of CPn-stimulated DCs T cell from aged subjects with their young counterparts.

Next, we determined the nature of cytokines secreted by T cells primed with CPn-stimulated DCs from aged and young subjects. Given the distinct profile of cytokines secreted by DCs from aged and young donors, we expected differences in the polarization of T helper (Th) cell responses towards Th_1_, Th_2_, Treg, Th_17_, or T_fh_. Indeed as is evident from Figure 5(a), culture of CPn-stimulated DCs from aged subjects induced significantly higher level (*P* = 0.03) of IFN-*γ* secretion from T cells as compared to their young counterparts. In contrast to IFN-*γ*, CPn-stimulated DCs from aged donors induced significantly reduced levels (*P* = 0.026, Figure 5(b)) of IL-10 production by T cells compared to young DC T cell coculture, further confirming that CPn-stimulated DCs from aged subjects display enhanced level of activation compared to DCs from young subjects. Secretion of IL-17 by T cells was comparable between aged and young subjects (Figure 5(c)). We also determined IL-21 secretion since IL-12 secretion by DCs can induce IL-21 in addition to IFN-*γ*. CPn-stimulated DCs from aged or young donors did not induce significant levels of IL-21 and the data was comparable in the two groups (Figure 5(d)). No IFN-*γ*, IL-10, IL-17, and IL-21 were detected in T cells cultured without DCs. DCs cultured alone without T cells for six days also did not secrete any of these cytokines (data not shown).

## 4. Discussion

The elderly population is reported to be at higher risk to develop respiratory illness such as COPD, bronchitis, and asthma [2, 3]. Several studies document the importance of persistent CPn infections in the pathogenesis of these diseases [7, 26]. However, the underlying mechanisms are not well understood. DCs are shown to be crucial, not only for sensitization to inhaled antigens, but also for establishing inflammation in the lung [27].* In vivo* studies have demonstrated that CPn can infect DCs and utilize them for dissemination from the lungs to other organs. Persistent CPn infection also induces a proinflammatory state in DCs [20, 21]. We and others have demonstrated that advancing age results in significant changes in the function of DCs. DCs from aged subjects are impaired in their capacity to control inflammation due to reduced production of anti-inflammatory cytokines, for example, IL-10 [22]. Here we report that age-associated alterations in DC functions enhance their response to CPn. These alterations induce an increased inflammatory response in the elderly as compared to young subjects.

Our results with monocyte-derived DCs are in keeping with previous studies which have reported that CPn infected DCs induce proinflammatory Th1/Th17 response via the production of IL-12, IL-6, and IL-1*β* [28, 29]. These studies all utilized viable CPn while our studies have been performed with UV inactivated CPn. The differences in the response between viable and UV inactivated bacteria are not apparent at the level of cytokine secretion by DCs since DCs can respond to bacterial components. However, there may be differences in other DC functions such as phagocytosis.

We did not observe differences in phenotype between DCs from aged and young subjects upon CPn stimulation. This is in agreement with previous reports where also phenotype of DCs from aged and young subjects after TLR stimulation was comparable [15, 30] but the secretion of inflammatory cytokines was elevated. The differences in cytokine production by DCs are not always associated with differences in phenotype [31]. In this study, also DCs from aged displayed an enhanced proinflammatory response upon CPn stimulation as is apparent from the increase in TNF-*α*, CXCL-10, and IL-12 and reduction in IL-10 secretion (Figures 2(a)–2(d)). We chose to assay CXCL-10 due to its important role as a key chemokine for the recruitment of Th1 lymphocytes into tissue and also because of the association of CXCL-10 with inflammatory and allergic diseases [32]. Furthermore, the infection of mice with CPn via the intranasal route also induced CXCL10 in the airways and enhanced inflammation [33]. Increased CXCL-10 secretion along with increased IL-12 by DCs from aged subjects will therefore enhance the Th1 inflammation in the airways of the elderly. In addition, increased TNF-*α* secretion by the DCs from aged subjects also favors Th1 responses. However, TNF secretion in response to CPn also inhibits the growth of the bacteria in DCs and regulates inflammation via induction of indoleamine 2,3-dioxygenase (IDO) [34]. The induction of IDO in DCs is a mechanism to prevent replication or persistence of CPn infection but since the present study was performed using UV inactivated CPn, we did not analyze the expression of IDO. Regulation of inflammation is impaired in DCs from aged subjects since IL-10 production in response to CPn is not elevated to counter the production of increased proinflammatory cytokines. This is in agreement with our previous observations which also demonstrated that stimulation of DCs from aged subjects with Lithium Chloride, which is well established IL-10 inducer in DCs, failed to do so in DCs from aged subjects suggesting that DCs from aged are inherently defective in the production of IL-10 [22].

The increase in inflammatory response is not due to altered TLR expression as we have previously reported that the expression of TLRs is comparable between aged and young DCs [30]. This has been further confirmed with our gene array data on aged and young DCs [35]. The gene array data also did not show any difference in the expression of TSLPR, IL-33R, or ICOSL on aged DCs as compared to young DCs [35]. However, we did observe alterations in the NF*κ*B signaling pathway which is similar to our previously reported findings [16] that DCs from aged subjects display an enhanced basal level of NF*κ*B activation [16], a major pathway involved in proinflammatory cytokine production from DCs. This enhanced NF*κ*B activation in DCs from aged subjects may be responsible for the increased secretion of inflammatory cytokines by aged DCs in response to TLRs and pathogens [22, 30, 36].

Interestingly, we also observed similar enhanced secretion of TNF-*α*, CXCL-10, and IL-6 and decreased IL-10 secretion from CPn-stimulated cDCs purified from the blood of the elderly (Figures 3(a)–3(d)) suggesting that increased inflammatory response to CPn is a feature of DCs from the elderly and is not due to the inflammatory nature of monocyte derived DCs [37].

In addition to IL-10, CPn-stimulated monocyte derived DCs from aged subjects were also deficient in production of innate interferons, IFN-*α* and IFN-*λ*, which serve as a primary host defense mechanism against infections (Figures 2(e) and 2(f)). CPn-stimulated cDCs from the blood of elderly also displayed a similar defect in the production IFN-*λ* (Figure 3(e)). IFN-*λ* is known to play an important role in the defense against respiratory tract infections, such as influenza A virus, respiratory syncytial virus, and SARS coronavirus [38]; however, its role in CPn infection has not been investigated. Our results suggest that IFN-*λ* may be an important protective cytokine in response to CPn. Previous reports from our laboratory have also demonstrated age-related impaired secretion of IFN-*α* and IFN-*λ* by plasmacytoid dendritic cells [23] and monocyte derived DCs during influenza infection [19]. This suggests that impairment in IFN secretion is an intrinsic age-related alteration and not associated with a specific type of infection.

Previous studies have reported the induction of IFN-*γ* and IL-17 producing CD4 T cells by CPn-stimulated DCs [28]. We also observed the secretion of both of these cytokines though the T cells stimulated with DCs from aged displayed enhanced proliferation and secreted increased levels of IFN-*γ* and decreased IL-10 compared to T cells stimulated with DCs from young subjects (Figures 4(a), 5(a), and 5(b)). The cytokines secreted by DCs in response to an infection dictate the proliferation and cytokine secretion of the T cells [39]. The secretion of IL-12 [31] and CXCL-10 [32] by DCs induces IFN-*γ* production in T cells and therefore increased production of these mediators by DCs from aged results in increased IFN-*γ* production while enhanced IL-12 and TNF by DCs from aged may be responsible for increased proliferation of T cells [13]. In addition, decreased IL-10 production by DCs from aged may further help increase proliferation and IFN-*γ* by T cells [13, 40]. A recent murine study has demonstrated that depletion of IL-10 producing T regulatory cells, which control inflammation and exacerbate airway inflammation and sensitization associated with CPn infection [41]. CD8 T cells are also reported to be necessary for clearing CPn infection in mice as depletion of CD8 T cells results in increased bacterial burden and infection kinetics [42]. Moreover, CD8 T cells also produce IFN-*γ* in response to chlamydial infection which complements the IFN-*γ* produced by CD4 T cells [43, 44]. Higher uncontrolled production of IFN-*γ* by both CD4 and CD8 T cells in aged subjects in response to CPn may therefore lead to aggravation of the inflammatory response in the airway of the aged population.

## 5. Conclusions

Altogether, our data suggest that DCs from aged are more activated and secrete higher level of inflammatory mediators in response to CPn but are still unable to produce a crucial cytokine such as IFN-*λ* involved in protection against respiratory infections. Increased inflammatory response of DCs from aged subjects to CPn induces an enhanced Th1 response in T cells which aggravates inflammation. CPn could therefore participate in promoting chronic airway inflammation in the elderly.

## Supplementary Material

The optimal concentration of CPn for activation of MoDCs was determined by stimulating the MoDCs with varying concentrations of CPn for 24h.Subsequently the DCs were collected and stained for upregulation of surface markers, CD83, CD86 by flow cytometry using specific antibodies. Supernatant collected was assayed for cytokines, TNF-a and IL1b by ELISA. Multiplicity of infection (MOI) 1CPn:1DC was found to be optimal. Bar diagrams depict the surface markers and cytokines in DCs after CPn stimulation. A. CD86; B. CD83; C. TNF-a; D. IL-1b.Figure is mean +/- S.D. of 5 subjects.

## Figures and Tables

**Table 1:** Description of the aged and young cohorts.

Age (range)	26 (20–32)	78 (65–93)
Gender, female	15 (62%)	18 (75%)
Comorbidities		
Osteoarthritis	0	12 (50%)
Hypertension	0	10 (41%)
Dyslipidemia	0	8 (33%)
Diabetes	0	0
Medications		
Vitamins	0	15 (62%)
Antioxidants	0	10 (41%)

Young *n* = 24 Aged *n* = 24.

## References

[1] Busse PJ, Lurslurchachai L, Sampson HA, Halm EA, Wisnivesky J (2010). Perennial allergen-specific immunoglobulin E levels among inner-city elderly asthmatics. *Journal of Asthma*.

[2] Diaz-Guzman E, Mannino DM (2010). Airway obstructive diseases in older adults: from detection to treatment. *Journal of Allergy and Clinical Immunology*.

[3] Reed CE (2010). Asthma in the elderly: diagnosis and management. *Journal of Allergy and Clinical Immunology*.

[4] Matsuse H, Tsuchida T, Fukahori S (2013). Differential airway inflammatory responses in asthma exacerbations induced by respiratory syncytial virus and influenza virus A. *International Archives of Allergy and Immunology*.

[5] McKenna JJ, Bramley AM, Skarbinski J, Fry AM, Finelli L, Jain S (2013). Asthma in patients hospitalized with pandemic influenza A(H1N1)pdm09 virus infection-United States, 2009. *BMC Infectious Diseases*.

[6] Burillo A, Bouza E (2010). Chlamydophila pneumoniae. *Infectious Disease Clinics of North America*.

[7] Sutherland ER, Martin RJ (2007). Asthma and atypical bacterial infection. *Chest*.

[8] Campbell LA, Kuo C-C (2002). *Chlamydia pneumoniae* pathogenesis. *Journal of Medical Microbiology*.

[9] Johnston SL, Martin RJ (2005). *Chlamydophila pneumoniae* and *Mycoplasma pneumoniae*: a role in asthma pathogenesis?. *American Journal of Respiratory and Critical Care Medicine*.

[10] Gnarpe J, Gnarpe H, Gause-Nilsson I, Lundorg P, Steen B (2000). Seroprevalence of antibodies to Chlamydia pneumoniae in elderly people: a two-decade longitudinal and cohort difference study. *Scandinavian Journal of Infectious Diseases*.

[11] Eddens T, Beaudoin S, Steinberger A (2012). Effect of age and vaccination on extent and spread of *Chlamydia pneumoniae* infection in C57BL/6 mice. *Immunity & Ageing*.

[12] Lambrecht BN, Hammad H (2012). Lung dendritic cells in respiratory viral infection and asthma: from protection to immunopathology. *Annual Review of Immunology*.

[13] Iwasaki A, Medzhitov R (2010). Regulation of adaptive immunity by the innate immune system. *Science*.

[14] Manicassamy S, Pulendran B (2009). Retinoic acid-dependent regulation of immune responses by dendritic cells and macrophages. *Seminars in Immunology*.

[15] Agrawal A, Gupta S (2011). Impact of aging on dendritic cell functions in humans. *Ageing Research Reviews*.

[16] Agrawal A, Tay J, Ton S, Agrawal S, Gupta S (2009). Increased reactivity of dendritic cells from aged subjects to self-antigen, the human DNA. *The Journal of Immunology*.

[17] Panda A, Qian F, Mohanty S (2010). Age-associated decrease in TLR function in primary human dendritic cells predicts influenza vaccine response. *The Journal of Immunology*.

[18] Agrawal A (2013). Mechanisms and implications of age-associated impaired innate interferon secretion by dendritic cells: a mini-review. *Gerontology*.

[19] Prakash S, Agrawal S, Cao J-N, Gupta S, Agrawal A (2013). Impaired secretion of interferons by dendritic cells from aged subjects to influenza—role of histone modifications. *Age*.

[20] Wantia N, Rodriguez N, Cirl C (2011). Toll-like receptors 2 and 4 regulate the frequency of IFN*γ*-producing cd4^+^ T-cells during pulmonary infection with *Chlamydia pneumoniae*. *PLoS ONE*.

[21] Beagley K, Huston WM, Hansbro PM, Timms P (2009). Chlamydial infection of immune cells: altered function and implications for disease. *Critical Reviews in Immunology*.

[22] Agrawal S, Gollapudi S, Gupta S, Agrawal A (2013). Dendritic cells from the elderly display an intrinsic defect in the production of IL-10 in response to lithium chloride. *Experimental Gerontology*.

[23] Sridharan A, Esposo M, Kaushal K (2011). Age-associated impaired plasmacytoid dendritic cell functions lead to decreased CD4 and CD8 T cell immunity. *Age*.

[24] Fulop T, Larbi A, Pawelec G (2013). Human T cell aging and the impact of persistent viral infections. *Frontiers in Immunology*.

[25] Lee N, Shin MS, Kang I (2012). T-cell biology in aging, with a focus on lung disease. * Journals of Gerontology Series A: Biological Sciences and Medical Sciences*.

[26] Blasi F, Damato S, Cosentini R (2002). *Chlamydia pneumoniae* and chronic bronchitis: association with severity and bacterial clearance following treatment. *Thorax*.

[27] Lambrecht BN, Hammad H (2003). Taking our breath away: dendritic cells in the pathogenesis of asthma. *Nature Reviews Immunology*.

[28] Flego D, Bianco M, Quattrini A (2013). *Chlamydia pneumoniae* modulates human monocyte-derived dendritic cells functions driving the induction of a Type 1/Type 17 inflammatory response. *Microbes and Infection*.

[29] Schröder NWJ, Crother TR, Naiki Y (2008). Innate immune responses during respiratory tract infection with a bacterial pathogen induce allergic airway sensitization. *Journal of Allergy and Clinical Immunology*.

[30] Agrawal A, Agrawal S, Cao J-N, Su H, Osann K, Gupta S (2007). Altered innate immune functioning of dendritic cells in elderly humans: a role of phosphoinositide 3-kinase-signaling pathway. *Journal of Immunology*.

[31] Agrawal S, Agrawal A, Doughty B (2003). Cutting edge: different Toll-like receptor agonists instruct dendritic cells to induce distinct Th responses via differential modulation of extracellular signal-regulated kinase-mitogen-activated protein kinase and c-Fos. *Journal of Immunology*.

[32] Liu M, Guo S, Hibbert JM (2011). CXCL10/IP-10 in infectious diseases pathogenesis and potential therapeutic implications. *Cytokine & Growth Factor Reviews*.

[33] Balogh EP, Faludi I, Virók DAP, Endrész V, Burián K (2011). *Chlamydophila pneumoniae* induces production of the defensin-like MIG/CXCL9, which has in vitro antichlamydial activity. *International Journal of Medical Microbiology*.

[34] Njau F, Wittkop U, Rohde M, Haller H, Klos A, Wagner AD (2009). In vitro neutralization of tumor necrosis factor-*α* during Chlamydia pneumoniae infection impairs dendritic cells maturation/function and increases chlamydial progeny. *FEMS Immunology and Medical Microbiology*.

[35] Cao J, Agrawal A, Jia Z, Sherman E, Gupta S (2014). Alterations in gene array patterns in dendritic cells from aged humans. *PLoS ONE*.

[36] Agrawal A (2010). Altered expression of NF*κ*B in ex vivo differentiated dendritic cells from the aged subjects: implications in immunotherapy. *Methods in Molecular Biology*.

[37] Neyt K, Lambrecht BN (2013). The role of lung dendritic cell subsets in immunity to respiratory viruses. *Immunological Reviews*.

[38] Mordstein M, Neugebauer E, Ditt V (2010). Lambda interferon renders epithelial cells of the respiratory and gastrointestinal tracts resistant to viral infections. *Journal of Virology*.

[39] Manicassamy S, Pulendran B (2009). Modulation of adaptive immunity with Toll-like receptors. *Seminars in Immunology*.

[40] Striz I, Brabcova E, Kolesar L, Sekerkova A (2014). Cytokine networking of innate immunity cells: a potential target of therapy. *Clinical Science*.

[41] Crother TR, Schröder NWJ, Karlin J (2011). *Chlamydia pneumoniae* infection induced allergic airway sensitization is controlled by regulatory T-cells and plasmacytoid dendritic cells. *PLoS ONE*.

[42] Penttilä JM, Anttila M, Varkila K (1999). Depletion of CD8^+^ cells abolishes memory in acquired immunity against *Chlamydia pneumoniae* in BALB/c mice. *Immunology*.

[43] Kyläniemi MK, Haveri A, Vuola JM, Puolakkainen M, Lahesmaa R (2009). Gene expression signatures characterizing the development of lymphocyte response during experimental Chlamydia pneumoniae infection. *Microbial Pathogenesis*.

[44] Rothfuchs AG, Kreuger MR, Wigzell H, Rottenberg ME (2004). Macrophages, CD4^+^ CD8^+^ cells are each sufficient for protection against *Chlamydia pneumoniae* infection through their ability to secrete IFN-*γ*. *The Journal of Immunology*.

