# Supplementary material for: Dendritic Cells from Aged Subjects Display Enhanced Inflammatory Responses to Chlamydophila pneumoniae
Source: Mediators Inflamm. 2014 Sep 1;2014:436438. doi: 10.1155/2014/436438 (PMC4165882; doi:10.1155/2014/436438)
Supplement: Supplementary file 1 — The optimal concentration of CPn for activation of MoDCs was determined by stimulating the MoDCs with varying concentrations of CPn for 24h.Subsequently the DCs were collected and stained for upregulation of surface markers, CD83, CD86 by flow cytometry using specific antibodies. Supernatant collected was assayed for cytokines, TNF-a and IL1b by ELISA. Multiplicity of infection (MOI) 1CPn:1DC was found to be optimal. Bar diagrams depict the surface markers and cytokines in DCs after CPn stimulation. A. CD86; B. CD83; C. TNF-a; D. IL-1b.Figure is mean +/- S.D. of 5 subjects. [file 436438.f1.pdf]

Supplementary Figure 1

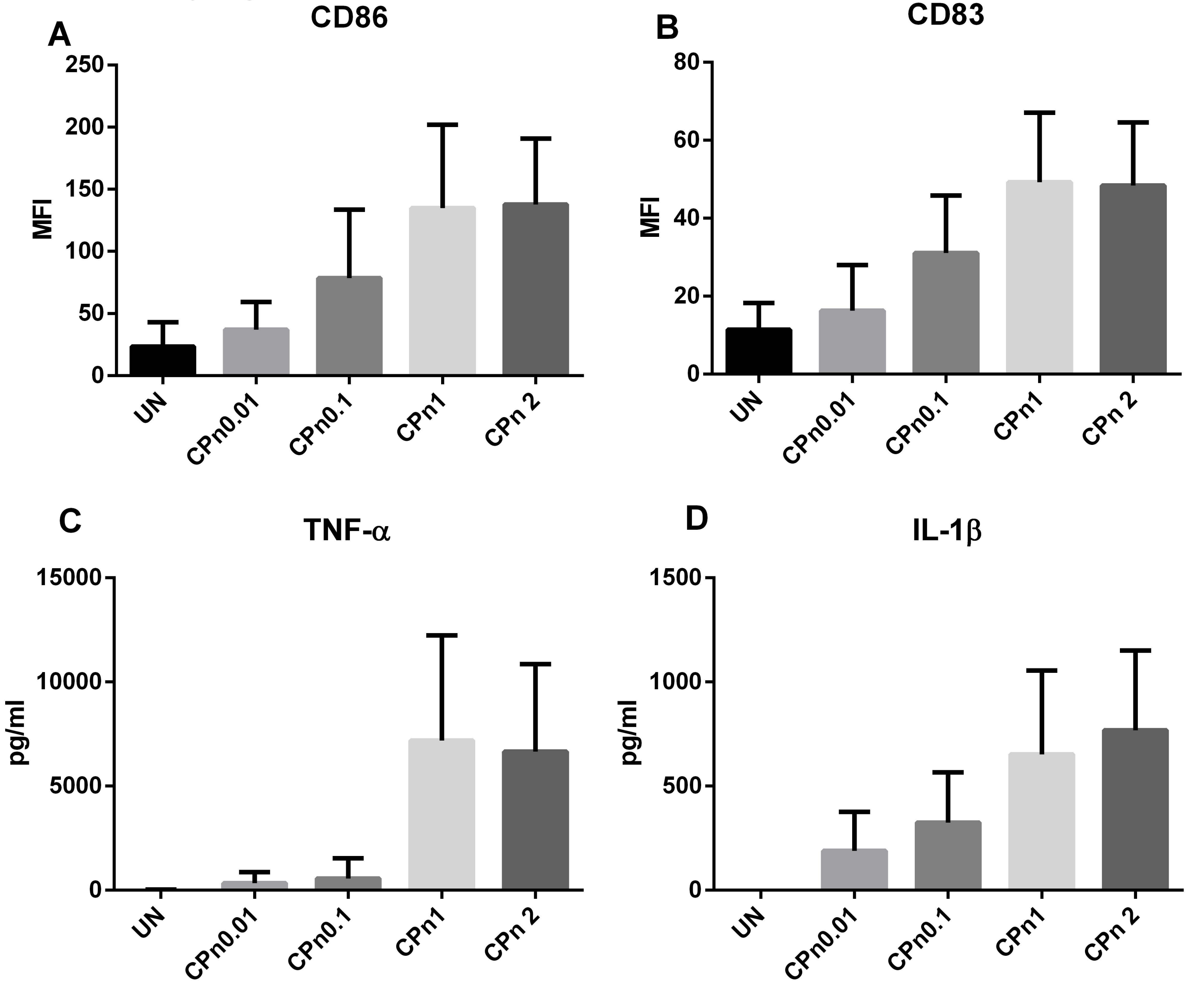

Supplementary figure 1: MOI of 1CPn:1DC is optimal. DCs were activated for 24h with varying MOIs of CPn. Upregulation of activation markers and concentrations of cytokines secreted was assayed. A. CD86 B. CD83 C. TNF-α D. IL-1β. Figure is mean +/-S.D. of 5 subjects.
